# A retrospective analysis of malaria epidemiological characteristics in Yingjiang County on the China–Myanmar border

**DOI:** 10.1038/s41598-021-93734-3

**Published:** 2021-07-08

**Authors:** Fang Huang, Shi-Gang Li, Peng Tian, Xiang-Rui Guo, Zhi-Gui Xia, Shui-Sen Zhou, Hong-Ning Zhou, Xiao-Nong Zhou

**Affiliations:** 1 National Institute of Parasitic Diseases, Chinese Center for Disease Control and Prevention; Chinese Center for Tropical Diseases Research; NHC Key Laboratory of Parasite and Vector Biology (National Institute of Parasitic Diseases, Chinese Center for Disease Control and Prevention); WHO Collaborating Centre for Tropical Diseases, National Center for International Research on Tropical Diseases, Shanghai, China; 2 Yingjiang County Center for Disease Control and Prevention, Yingjiang, Yunnan China; 3 Yunnan Institute of Parasitic Diseases, Pu’er, Yunnan China

**Keywords:** Epidemiology, Infection

## Abstract

Yingjiang County, which is on the China–Myanmar border, is the main focus for malaria elimination in China. The epidemiological characteristics of malaria in Yingjiang County were analysed in a retrospective analysis. A total of 895 malaria cases were reported in Yingjiang County between 2013 and 2019. The majority of cases occurred in males (70.7%) and individuals aged 19–59 years (77.3%). *Plasmodium vivax* was the predominant species (96.6%). The number of indigenous cases decreased gradually and since 2017, no indigenous cases have been reported. Malaria cases were mainly distributed in the southern and southwestern areas of the county; 55.6% of the indigenous cases were reported in Nabang Township, which also had the highest risk of imported malaria. The “1–3–7” approach has been implemented effectively, with 100% of cases reported within 24 h, 88.9% cases investigated and confirmed within 3 days and 98.5% of foci responded to within 7 days. Although malaria elimination has been achieved in Yingjiang County, sustaining elimination and preventing the re-establishment of malaria require the continued strengthening of case detection, surveillance and response systems targeting the migrant population in border areas.

## Introduction

Malaria, an infectious disease, has had the longest epidemic duration, and the broadest impact in the history of China^1^. It has been estimated that there were at least 30 million malaria cases annually before 1949^2^. Following the implementation of a significant integrated malaria control and elimination programme for more than seven decades and specifically the “1–3–7” surveillance and response strategy that was developed and has been implemented since 2010^3,4^, the disease burden has been greatly reduced^2,5^. Since 2017, no indigenous cases have been reported, achieving the standard of national malaria elimination^6^. Currently, China is ready for nationwide malaria elimination certification by the World Health Organization (WHO).

Yunnan Province borders Myanmar, Laos, and Vietnam; the border extends 4060 km, touching 25 counties, with 17 border ports, and 643 border crossings. There is no natural barrier along the border^7^. According to malaria epidemiological data collected from surveillance systems in China's border areas, the population crossing the border and border residents of Yunnan Province, China, have a higher risk of malaria transmission^8^. Historically, malaria has been serious in Yunnan Province with more than 400 thousand malaria cases^9,10^. Since 2010, Yunnan has been the only province with *Plasmodium falciparum* transmission in China^11^. The last indigenous malaria case in China was reported in Yingjiang County of Yunnan Province in 2016^6^; there have been no indigenous cases reported nationwide since, which allowed Yunnan Province to achieve verification of malaria elimination in June 2020.

A total of 18 counties in Yunnan Province sit on the China–Myanmar border. One of them, Yingjiang County, is located in western Yunnan Province and having a border length of 214.6 km; it borders Kachin State, Myanmar, in the western region^10^. The border areas on both sides are rural, hard-to-reach, poverty-stricken areas inhabited by minority nationalities^12,13^. The county has nine townships bordering Myanmar, five entry-exit highways, two provincial ports, four passages, and 33 boundary passages^14^. The climate, landscape and vectors of malaria on both sides of the border are similar^12,13^. In the last 10 years, the incidence of malaria in Yingjiang County has declined remarkably and this progress was made possible through greater access to effective malaria control tools. The last indigenous malaria case in China was caused by *P. vivax* infection in April 2016, located in Taiping Township of Yingjaing County^6^.

On the Myanmar side of the border with Yingjiang County, is Laiza, Kachin State (see Additional file 1), with a population of approximately 40,000; the area has been affected by malaria epidemics for several decades^15^. According to a malaria report from the China–Myanmar Joint Prevention and Control Information Exchange Mechanism, thousands of malaria cases were reported in Laiza in 2016 and 2017. However, the number of malaria cases has decreased dramatically in the last three years, supported by cross-border cooperation to achieve malaria elimination, timely case detection and appropriate treatment, and vector control and surveillance. The number of cases increased in 2020 as an effect of the COVID-19 pandemic (see Additional file 2). In September 2019, nearly 200 cases of vivax malaria were detected in a resettlement site containing 2500 residents on the Myanmar side of the border with Nabang Township of Yingjiang County (unpublished data).

Human population movement is one of the significant challenges in malaria control and elimination, especially in border areas. Population movement from high to low or non-malaria-endemic areas can result in imported infections, which may promote further transmission and threaten the success of previous elimination efforts^16^. Although malaria elimination has been achieved on the Chinese side of the border, the high risk of importation, introduction and re-establishment of malaria in the post-elimination stage should be of concern because of the migrant population crossing the border and the lack of barriers for malaria vectors^17^. Therefore, we conducted an epidemiological analysis of malaria in Yingjiang County to investigate the epidemiological characteristics of malaria and to identify the risk of re-establishment of malaria in this border area in the post-elimination stage.

## Results

A total of 895 malaria cases were reported in the entire county in 2013–2019, including 45 indigenous cases and 850 imported cases. All cases were diagnosed by microscopy, and except 7, were also confirmed by polymerase chain reaction (PCR). There were no malaria-associated deaths reported during this period. The number of malaria cases was the lowest, at 72, in 2013 and reached a peak of 186 in 2016; then, it decreased to 92 in 2019. Malaria cases occurred predominantly in males (males 70.7%, n = 633; females 29.3%, n = 262), with a sex ratio of 2.42:1, but the difference was not significant (*P* = 0.2093). The majority of cases occurred in individuals aged 19–59 years (77.3%, 692/895). Most patients were outdoor workers (56.5%, 506/895), who have the highest risk of malaria infection (*P* < 0.0001); while indoor workers accounted for 27.8% (249/895) of the cases.

The proportion of indigenous malaria cases decreased from 25.0% (18/72) in 2013 to 0.05% (1/185) in 2016. The last indigenous malaria case reported in 2016 in Yunnan Province, was also the last in the whole country. Since 2017, no indigenous cases have been reported. *P. vivax* was the predominant malaria parasite in Yingjiang County. In 2013–2019, the proportion of *P. vivax* malaria was 96.6% (range: 89.73–100.0%). *P. vivax* infection was the most common cause of malaria in Yingjiang County, as well as in Yunnan Province. The annual reported number of *P. falciparum* cases was less than 10 from 2013–2019, and no *P. falciparum* cases was reported in 2019. One *P. malariae* infection and two mixed infections involving *P. falciparum* and *P. vivax* were reported (Table 1).

**Table 1:** Demographic characteristics of the reported malaria cases in Yingjiang County, 2013–2019.

Demographic characteristics	2013	2014	2015	2016	2017	2018	2019	Total	*P* value
	Number (%)	Number (%)	Number (%)	Number (%)	Number (%)	Number (%)	Number (%)	Number (%)	
**Sex**									
Male	48(66.7)	70(80.5)	131(74.9)	124(67.0)	121(67.6)	72(68.6)	67(72.8)	633(70.7)	0.2039
Female	24(33.3)	17(19.5)	44(25.1)	61(33.0)	58(32.4)	33(31.4)	25(27.2)	262(29.3)	
**Age**									
< 5 years	0	5(5.7)	6(3.4)	8(4.3)	8(4.5)	4(3.8)	1(1.1)	32(3.6)	0.0216*
5–18 years	14(19.4)	9(10.3)	20(11.4)	27(14.6)	30(16.8)	17(16.2)	3(3.3)	120(13.4)	
19–59 years	56(77.8)	71(81.6)	141(80.6)	139(75.1)	131(73.2)	73(69.5)	81(88.0)	692(77.3)	
≥ 60 years	2(2.8)	2(2.3)	8(4.6)	11(5.9)	10(5.6)	11(10.5)	7(7.6)	51(5.7)	
**Occupation**									
Outdoor workers ^a^	56(77.8)	76(87.4)	115(65.7)	105(56.8)	74(41.3)	42(40.0)	38(41.3)	506(56.5)	< 0.0001*
Indoor workers ^b^	7(9.7)	3(3.4)	31(17.7)	57(30.8)	80(44.7)	42(40.0)	29(31.5)	249(27.8)	
Unclear^**#**^	8(11.1)	8(9.2)	23(13.1)	22(11.9)	21(11.7)	21(20.0)	25(27.2)	128(14.3)	
Missing	0	0	6(3.4)	1(0.5)	4(2.2)	0	0	11(1.2)	
**Plasmodium species**									
*P. vivax*	66(91.7)	78(89.7)	174(99.4)	182(98.4)	173(96.6)	100(95.2)	92(100.0)	865(96.6)	< 0.0001*
*P. falciparum*	6(8.3)	9(10.3)	1(0.6)	3(1.6)	6(3.4)	2(1.9)	0	27(3.0)	
*P. malariae*	0	0	0	0	0	1(1.0)	0	1(0.1)	
Mix infection^§^	0	0	0	0	0	2(1.9)	0	2(0.2)	
**Case classification**									
Imported cases	54(75.0)	67(77.0)	169(96.6)	184(99.5)	179(100.0)	105(100.0)	92(100.0)	850(95.0)	< 0.0001*
Indigenous cases	18(25.0)	20(23.0)	6(3.4)	1(0.5)	0			45(5.0)	
**Level of health facility providing the diagnosis health facility**									
County CDC	14(19.4)	35(40.2)	14(8.0)	20(10.8)	12(6.7)	7(6.7)	10(10.9)	112(12.5)	< 0.0001*
County hospital	31(43.1)	20(23.0)	23(3.1)	33(17.8)	20(11.2)	24(22.9)	26(28.3)	177(19.8)	
Township hospital	24(33.3)	29(33.3)	135(77.1)	128(69.2)	141(78.8)	73(69.5)	50(54.3)	580(64.8)	
Village clinic	2(2.8)	1(1.1)	2(1.1)	0	0	1(1.0)	5(5.4)	11(1.2)	
Private clinic	1(1.4)	2(2.3)	0	1(0.5)	0	0	1(1.1)	5(0.6)	
Missing	0	0	1(0.6)	3(1.6)	6(3.4)	0	0	10(1.1)	
Total	72	87	175	185	179	105	92	895	

^a^Outdoor workers were persons whose activities were mostly conducted outside and had a high risk of exposure to outdoor biting vectors, especially outdoor night-time workers. This included Architectural engineers, Construction workers, Farmers, Fishermen, Open mine workers, Sailors/Truck drivers, Field engineers, Herdsmen, Militaries/Soldiers, etc.

^b^Indoor workers were persons who worked mostly indoors and had a low risk of exposure to outdoor biting vectors. This included Businessmen, Caterers, Interpreters, Medical staff, Office workers, Teachers, Actors, Flight attendants, Baby-sitters, Middlemen, Cooks, Diplomats, Financial staff, Journalists, Underground mine workers, Prisoners, Researchers, Waiters, etc.

^#^Unclear indicates that the risk exposure could not be estimated in populations such as Children, Retirees, Students, Unemployed persons, etc. Missing data were not included in the statistical analysis.

^§^Mixed infection refers to simultaneous *P. falciparum* and *P. vivax* infection. *Fisher's exact test was used to evaluate differences among the groups if 25% of the cells had expected counts less than 5.

**Figure 1:**
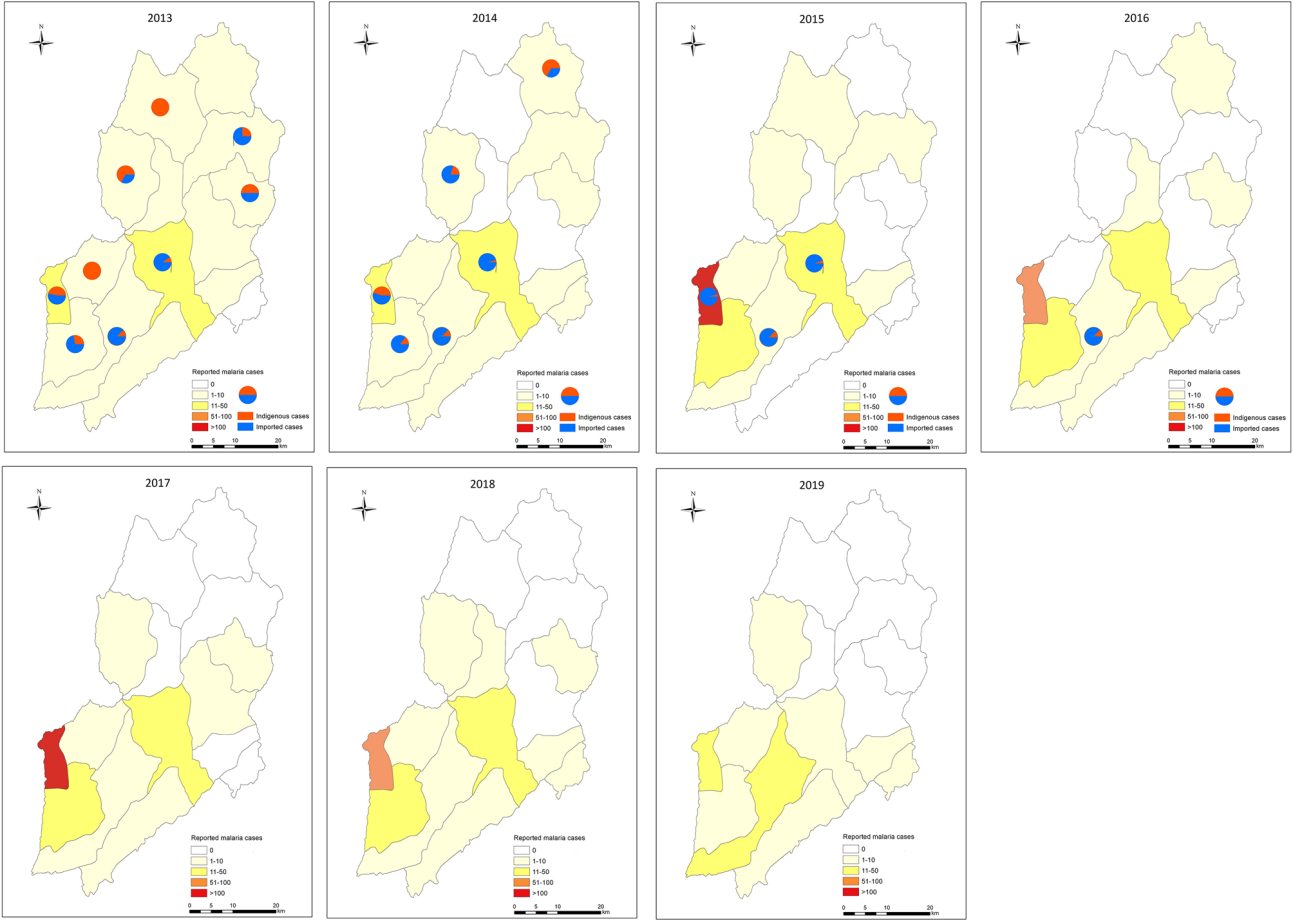
The number of reported malaria cases in 15 townships and the proportion of indigenous and imported cases at the township level. Townships was colored based on the number of reported malaria cases in each year. The pie indicates the proportion of indigenous cases and imported cases in the townships with indigenous cases reported in 2013–2016. Maps were created by the first author (FH) using ArcGIS version 10.1, https://www.arcgis.com, and data of malaria cases were from publicly available sources (CIDRS, Malaria Elimination Programme, China).

There are 15 townships in Yingjiang County and nine of which border Myanmar (Fig. 1). Most malaria cases were distributed in the southern and southwestern parts of the county. In 2013–2019, 54.0% (483/895) of the malaria cases were reported from Nabang Township, followed by Pingyuan Township, where the local government of Yingjiang County is located, with 15.5% (139/895). In 2013, a total of 72 malaria cases were reported in Yingjiang County; the cases were distributed in 14 out of 15 townships, while only six townships reported malaria cases in 2019. The number of reported malaria cases decreased slightly at the township level over time. In addition, the number of townships with indigenous cases decreased from eight in 2013, to six in 2014, three in 2015 and only one in 2016 (Fig. 1). Among all 45 indigenous cases, 55.6% occurred in Nabang Township, which also had the highest risk of imported malaria. The last indigenous malaria case was reported in Taiping Township in April 2016, which was also the last indigenous malaria case reported in the whole country. Since 2017, no indigenous cases have been reported in China, including these border counties.

**Figure 2:**
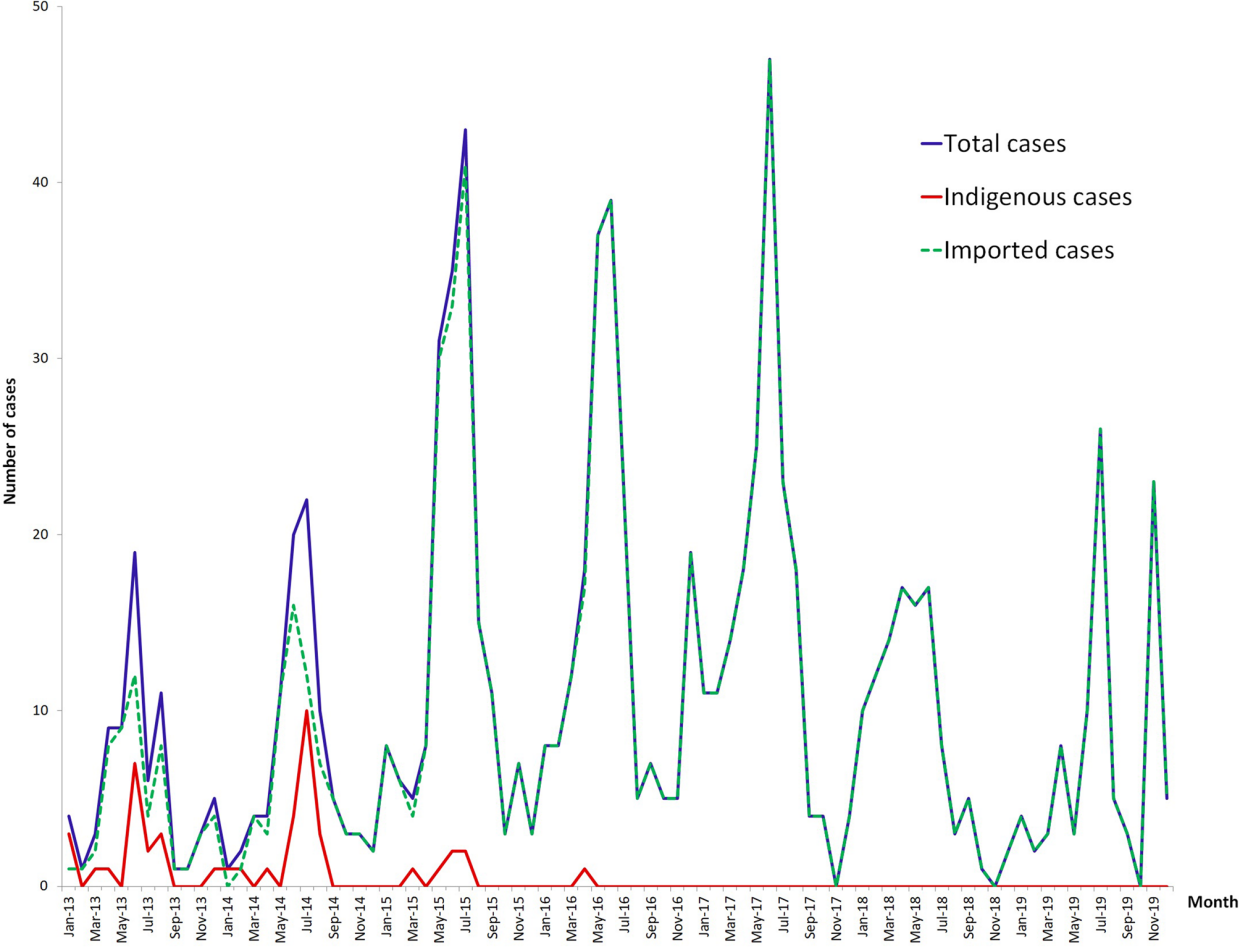
Monthly distribution of malaria cases in Yingjiang County, 2013–2019. The number of imported, indigenous and total cases were displayed monthly. The line of imported cases and total cases were overlap from May of 2016 to December of 2019.

The number of reported malaria cases in Yingjiang County displayed well-defined seasonality in 2013–2017, with one peak from May to July and a second smaller peak from November to the following January (Fig. 2). This seasonality was mainly associated with the geographical environment and meteorological factors that are strongly correlated with the abundance of *Anopheles*. There are only two seasons in the border area of Yingjiang County, the rainy season from May to September and the dry season from October to the following April. In 2018, the number of malaria cases reported from May to July decreased with a lower peak than that observed in previous years. In 2019, there were two similar peaks in June to July and in November (Fig. 2). The monthly trend was mainly caused by imported malaria cases among the migrant population not the natural dynamics of malaria transmission because only imported malaria cases have been reported since 2017.

**Figure 3:**
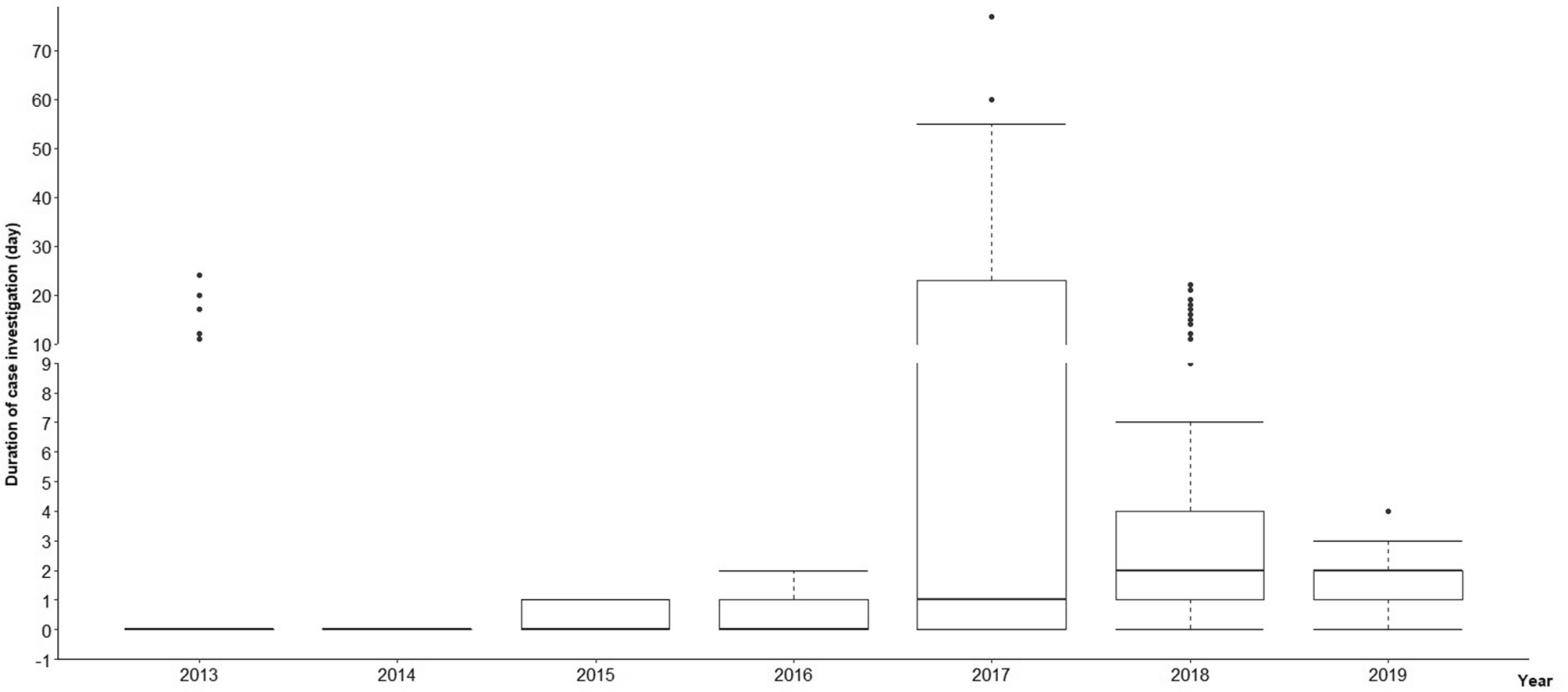
Durations of case investigations and confirmations by county CDCs, 2013–2019. The median, range and upper and lower quartiles, and discrete values of the data were divided by discrete duration ranges. The figure was created in R using ggplot2 package (R Core Team, Vienna, Austria, 2020, www.R-project.org; version 4.0.3).

The “1–3–7” approach describes a system of activities dependent on timely diagnosis and response to malaria cases; the strategy includes strict hourly timelines to be followed. Within 24 h, any malaria case must be reported. By the end of day 3, the county’s Center for Disease Control and Prevention (CDC) must confirm and investigate the case and determine whether there is a risk of spread. By the end of day 7, the county CDC must perform measures or interventions to prevent further spread by testing residents and family members in the community where the malaria case was identified. From 2013 to 2019, there were no delays in the reporting of malaria cases, and 895 malaria cases in Yingjiang County were reported within 1 day. In terms of case investigation and confirmation, an average of 88.9% (range: 64.2–100%) of cases were investigated within 3 days, and this indicator reached 100% in 2014, 2015, and 2016 (Fig. 3). The median case investigation durations from 2013 to 2019 were 0, 0, 1, 1, 33, 3, and 2 days, respectively, and the interquartile range (IQRs) of durations in each year are shown in Fig. 3. There were significant differences between years (*P* < 0.001). A total of 261 foci were identified during the study period, and 98.5% (256/261) were investigated and responded to within 7 days.

Of the 895 reported malaria cases, 97.1% (n = 869) were diagnosed in a health facility of a county CDC/hospital or township hospital (Table 1). The proportion of cases diagnosed at township hospitals increased slightly, while those diagnosed at county-level health facilities remained at a low level, indicating that relying on the capabilities of township hospitals is key.

## Discussion

In 2010, China set an ambitious goal to eliminate malaria by 2020 and established a cooperative agreement among 13 ministries to eliminate malaria nationwide^1^. The “1–3–7” surveillance and response strategy, introduced after China launched the national malaria elimination programme, successfully reduced the number of indigenous cases of malaria to zero, where it has remained since 2017^6^. Additionally, Yunnan Province, which shares borders with the malaria-endemic countries of Lao People’s Democratic Republic, Myanmar and Vietnam, has achieved malaria elimination^18,19^.

Yingjiang County is one of 18 counties along the China–Myanmar border and has the highest risk of malaria transmission in Yunnan Province. According to the classification of the Malaria Elimination Action Plan (2010–2020), Yingjiang County was a Type I county (with indigenous cases reported for 3 years and an incidence rate ≥ 1/10,000) that was targeted for the implementation of key strategies to strengthen surveillance and reduce the incidence in the pre-elimination stage^4^. This study showed that malaria cases in Yingjiang County decreased significantly, with a total of 895 cases reported from 2013 to 2019, and no indigenous malaria cases reported on the Chinese side of the border in the past three years. The decline in indigenous cases on the Chinese side of the border was primarily attributed to the "1–3–7" surveillance and quick response strategy, which was not implemented on the Myanmar side. However, the reduction in imported cases in Yingjiang County was highly associated with malaria transmission in Kachin state, Myanmar, because 99% of imported malaria cases were imported from Myanmar. In the past several years, the malaria burden has been reduced in Kachin state due to cooperation between China and Myanmar regarding malaria elimination. However, the risk of malaria re-establishment in Chinese border areas should be of concern because malaria transmission still occurs on the Myanmar side^13,20^. Different cross-border activities may result in different temporal patterns of imported cases (e.g., the number of peak transmission seasons). For example, people engaged in frontier trade may frequently cross the border, while local farmers may go to Myanmar for logging or mining during the slack seasons in farming. In November 2019, the Hongbenghe Water Conservancy Construction Site, which was located at the China–Myanmar border in Taiping Township of Yingjiang County, was identified as an epidemic focus, and a total of 22 *P. vivax* malaria cases were identified. According to the case investigation, the source of infection was the bite of an *Anopheles* mosquito carrying malaria parasites that was transported from overseas; therefore, these cases were classified as imported malaria cases. This epidemic cluster caused another peak in monthly malaria cases in Yingjiang in 2019 (Fig. 2). Nabang Township, which reported more than half of the malaria cases in the county, borders Laiza in Kachin State of Myanmar. There is one provincial port and several boundary passages in this township and hundreds of migrants cross the border every day. According to local immigration records, approximately fifty thousand border crossings occur monthly at Nabang Port. This migrant population in the rural areas was less educated in preventive measures along with constantly low level of the community’s knowledge, attitude and practice (KAP) regarding malaria. Moreover, some sections of the border comprise a stream that is only a few meters wide, and mosquitoes carrying parasites can cross the border easily. Similar geographical environments and meteorological factors along the border area play major roles in malaria transmission, mostly by mediating effects on both the mosquito vector and the development of the malaria parasite inside the mosquito vector^23^. The species of malaria vectors in Yunnan Province are the most diverse in China. According to a vector survey, a total of 11 *Anopheles* species have been identified in Nabang and Tongbiguan townships of Yingjiang County. *Anopheles minimus* is the primary malaria vector, followed by *An. sinensis*^14,23,24^.

*P. vivax* is the most geographically widespread parasite causing human malaria, and over 2.5 billion people are at risk of infection^25,26^. *P. vivax* malaria is mainly endemic in Southeast Asia and Central and South America. As reported by the WHO, more than half of *P. vivax* infections (53%) occur in the WHO South-East Asia region^12,27^. The proportion of vivax malaria cases in Yingjiang County increased from 2013 to 2019 and no falciparum malaria cases were reported in 2019, indicating that *P. vivax* was the predominant species on the Myanmar side and the rate of *P. falciparum* infection in Myanmar was very low because more than 95% of the imported cases were from Myanmar. This change was similar to that in other countries in the Greater Mekong sub-region^28,29^. The epidemiology of vivax malaria in this region is highly heterogeneous and *P. vivax* has become a major challenge to malaria elimination in this region^30,31^.

China’s “1–3–7” surveillance and response strategy was developed and used for malaria elimination nationwide^2,3^. In this study, we analysed the three indicators included in the “1–3–7” approach in Yingjiang County. All malaria cases were reported to the CIDRS within 24 h, indicating that the web-based case reporting system is sensitive, as it allowed a prompt response, even in township hospitals. The proportion of case investigations and confirmations completed within 3 days was 88.9%, compared with only 64.2% in 2017 (Fig. 3). The main reasons for the lower rate were that some patients were located in very remote and hard-to-reach areas; therefore, it took the county CDC longer than usual to complete the case investigation and confirmation. During the study period, 98.5% (256/261) of the foci were investigated and responded to within 7 days. The “1–3–7” approach was implemented effectively in Yingjiang County at the county level, similar to other counties^32^. This strategy is not only the technical specification for malaria elimination in China, but it has been adopted by the WHO, and is included in the *Malaria surveillance monitoring & evaluation, a reference manual*^33^. Recently, the “1–3–7” approach has been popularized and applied in many countries and regions worldwide and has made great contributions in the progress toward global malaria elimination^34–36^.

Although great achievements have been made in reducing the overall number of cases of malaria and eliminating indigenous cases in China, the elimination campaign faces challenges in areas near international borders^22,37^. The specific environmental, administrative, anthropological, and geographic characteristics of border areas have a unique impact on the epidemiology of malaria. Cross-border malaria is difficult to manage because of political, economic and geographic constraints^38,39^. In addition, the coronavirus disease 2019 (COVID-19) pandemic has affected the availability of key malaria control interventions and may cause malaria-associated morbidity and mortality to increase in the future^40,41^.

## Conclusion

Substantial gains have been made in reducing the number of malaria cases and eliminating indigenous malaria in Yingjiang County. Sustaining elimination and preventing the re-establishment of malaria require the strengthening of case detection, surveillance and response system targeting migrant populations in border areas.

## Methods

### Description of the study area

**Figure 4:**
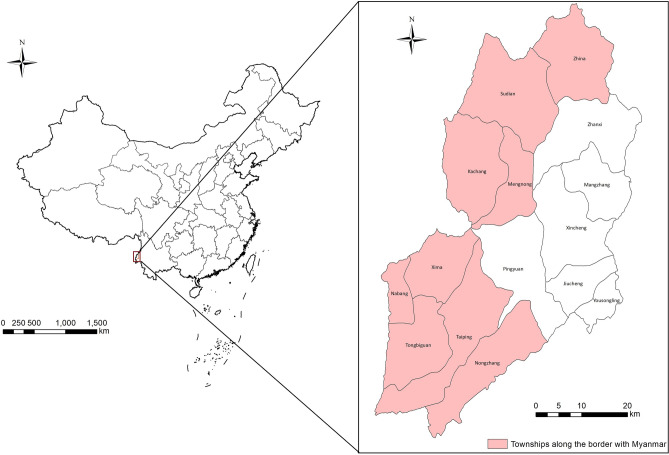
Location of Yingjiang County and the townships along the China–Myanmar border. A total of 15 townships were included in Yingjiang County, nine of which borders Myanmar shown with light red color. Maps were created by the first author (FH) working with ArcGIS version 10.1, https://www.arcgis.com.

Yingjiang County is located in western Yunnan Province (97°31′–98°16′N, 24°24′–25°20′E). It shares a long border with Kachin State, Myanmar and had the highest risk of malaria transmission in Yunnan Province (Fig. 4)*.* The land area of Yingjiang County is 4429 km^2^, with a local population of 316,990 people. Yingjiang County is mountainous with several alluvial plains. The county has various climate types, ranging from tropical to subtropical and temperate zones, and has an average annual temperature of 22.7 °C and annual rainfall of 2.65 m. Intact forests exist in the mountains above 2000 m. The elevation varies from 210 to 3404 m. The county is within a very active seismic zone and was affected by violent earthquakes in 2008, 2009 and 2011. Migration, plantation and logging activities are frequent along the border^42^. *An. sinensis* and *An. minimus* are reported to be the dominant vectors for malaria parasites^23,43^.

### Data collection

Malaria case data and demographic information, including sex, age, occupation, residential address, type of disease, date of onset, and date of confirmation were collected from the web-based Chinese Infectious Disease Report System (CIDRS) from 2013 to 2019^44,45^. Malaria cases were classified according to the criteria of the national guidelines of China as laboratory-confirmed malaria, which referred to patients with a positive result on one type of laboratory test, including microscopy, a rapid diagnostic test (RDT), or PCR, or clinically diagnosed malaria, which referred to patients with malaria–like signs and symptoms but without the detection of parasites on blood examination^46^. Malaria case data from 15 townships were extracted to map the malaria distribution at the township level. In addition, according to the Technical Program for Malaria Elimination in China^47^, indigenous malaria was defined as malaria infection obtained from within the province in which the diagnosis was made, while imported malaria was defined as malaria whose origin can be traced to a transmission area outside the province in which the malaria diagnosis of malaria was made; moreover, the following criteria for imported malaria was also applied: the patient has received a malaria diagnosis; the patient had a travel history to a malaria-endemic area outside China during the malaria transmission season; and the onset time for malaria was less than 1 month after returning to China during the local transmission season.

### Data analysis

R software (Version 4.0.3; R Core Team, Vienna, Austria, 2020; www.R-project.org)^48^ and SAS software (SAS Institute Inc, Version 9.2, Cary, NC, USA) were used for data processing and statistical analysis. The chi-squared test was used to evaluate differences among the different sub-groups; Fisher's exact test was used if 25% of the cells had expected counts less than 5. Maps were created using ArcGIS 10.1 (Environmental Systems Research Institute, Inc, Redlands, CA, USA). The indicators used to evaluate the implementation of the “1–3–7” approach were determined from the individual case reports downloaded from the CIDRS. A *P* value < 0.05 was considered statistically significant.

### Ethical approval

This study has been approved by the Ethical Review Committee of National Institute of Parasitic Diseases, Chinese Center for Disease Control and Prevention, No. 2019008.

## Supplementary Information

Supplementary Information 1.

Supplementary Information 2.

## Data Availability

The data used in this study is publicly available.
