# Supplementary material for: A retrospective analysis of malaria epidemiological characteristics in Yingjiang County on the China–Myanmar border
Source: Sci Rep. 2021 Jul 8;11:14129. doi: 10.1038/s41598-021-93734-3 (PMC8266812; doi:10.1038/s41598-021-93734-3)
Supplement: Supplementary file 1 — Supplementary Information 1. [file 41598_2021_93734_MOESM1_ESM.docx]

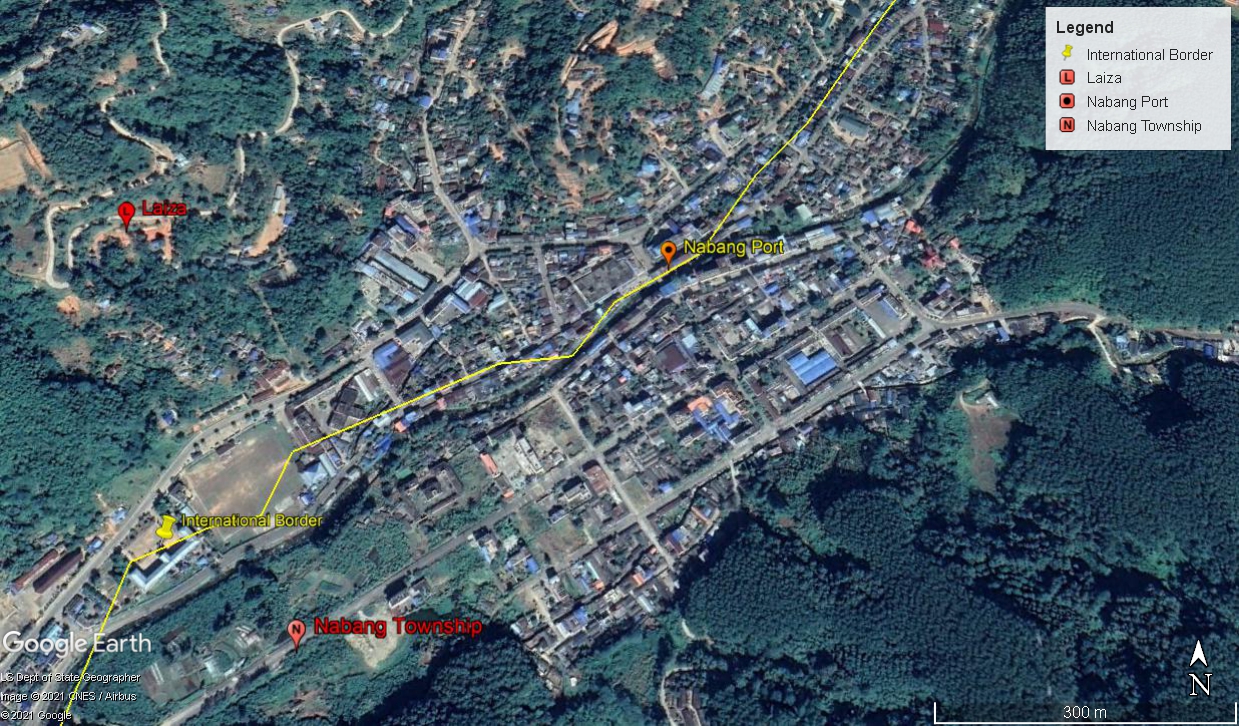


**File 1.** Satellite image of border area between Yingjiang County, Yunnan Province and Laiza, Kachin State. The map was prepared by the first author (FH) using Google Earth Pro, version 7.3.3.7786, 2020, https://www.google.com/earth/versions/.
