# Supplementary figures and images for: A retrospective analysis of malaria epidemiological characteristics in Yingjiang County on the China–Myanmar border
Source: Sci Rep. 2021 Jul 8;11:14129. doi: 10.1038/s41598-021-93734-3 (PMC8266812; doi:10.1038/s41598-021-93734-3)

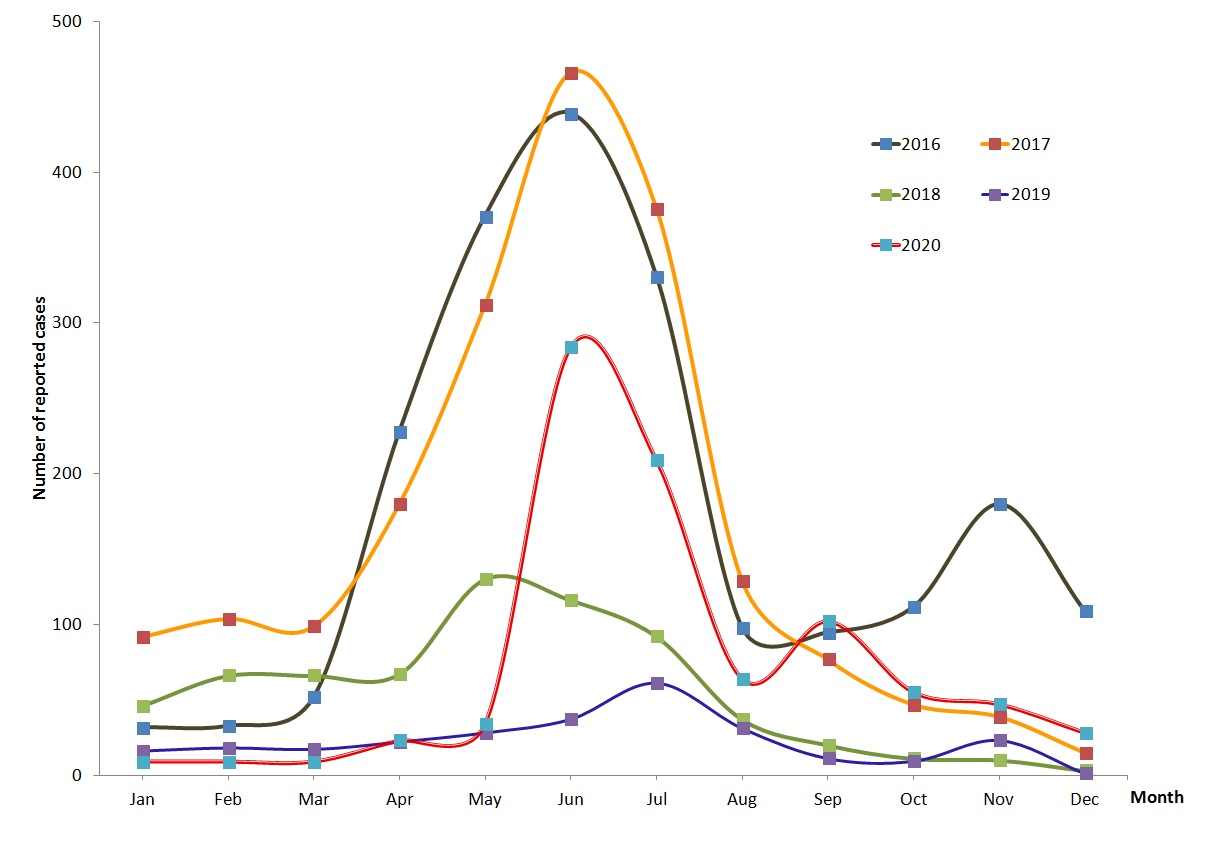


**File 2.** Reported cases of malaria from the sentinel sites in Kachin State (Special Region 2), Myanmar, 2016**–**2020.

Supplement: Supplementary file 2 — Supplementary Information 2. [file 41598_2021_93734_MOESM2_ESM.docx]
